# The evolution of the mitochondrial disease diagnostic odyssey

**DOI:** 10.1186/s13023-023-02754-x

**Published:** 2023-06-22

**Authors:** John L. P. Thompson, Amel Karaa, Hung Pham, Philip Yeske, Jeffrey Krischer, Yi Xiao, Yuelin Long, Amanda Kramer, David Dimmock, Amy Holbert, Cliff Gorski, Kristin M. Engelstad, Richard Buchsbaum, Xiomara Q. Rosales, Michio Hirano

**Affiliations:** 1 Department of Biostatistics, Mailman School of Public Health, Columbia University Irving Medical Center, New York, USA; 2 Division of Genetics, Massachusetts General Hospital/Harvard Medical School, Boston, MA USA; 3 United Mitochondrial Disease Foundation, Pittsburgh, PA USA; 4 University of South Florida Health Informatics Institute, Tampa, FL USA; 5 Department of Population and Family Health, Mailman School of Public Health, Columbia University, New York, USA; 6 Creyon Bio, San Diego, CA USA; 7 Tampa General Hospital, Tampa, FL USA; 8 Department of Neurology, Columbia University Irving Medical Center, New York, USA

## Abstract

**Background:**

Mitochondrial diseases often require multiple years and clinicians to diagnose. We lack knowledge of the stages of this diagnostic odyssey, and factors that affect it. Our goals are to report the results of the 2018 Odyssey2 (OD2) survey of patients with a medical diagnosis of mitochondrial disease; and to propose steps to reduce the odyssey going forward, and procedures to evaluate them.

**Methods:**

Data are from the NIH-funded NAMDC-RDCRN-UMDF OD2 survey (N = 215). The main outcomes are Time from symptom Onset to mitochondrial disease Diagnosis (TOD) and Number of Doctors Seen during this diagnostic process (NDOCS).

**Results:**

Expert recoding increased analyzable responses by 34% for final mitochondrial diagnosis and 39% for prior non-mitochondrial diagnosis. Only one of 122 patients who initially saw a primary care physician (PCP) received a mitochondrial diagnosis, compared to 26 of 86 (30%) who initially saw a specialist (*p* < 0.001). Mean TOD overall was 9.9 ± 13.0 years, and mean NDOCS 6.7 ± 5.2. Mitochondrial diagnosis brings extensive benefits through treatment changes and increased membership in and support of advocacy groups.

**Conclusions:**

Because TOD is long and NDOCS high, there is great potential for shortening the mitochondrial odyssey. Although prompt patient contact with primary mitochondrial disease specialists, or early implementation of appropriate tests, may shorten the diagnostic odyssey, specific proposals for improvement require testing and confirmation with adequately complete, unbiased data across all its stages, and appropriate methods. Electronic Health Record (EHRs) may help by accessing diagnostic codes early, but their reliability and diagnostic utility have not been established for this group of diseases.

**Supplementary Information:**

The online version contains supplementary material available at 10.1186/s13023-023-02754-x.

## Background

Mitochondria are vital cellular organelles that generate most of the cellular energy in the form of adenosine triphosphate (ATP) (see Glossary, Table 1) via oxidative phosphorylation (OxPhos) [1]. Virtually all human cells require mitochondria to function [2]. As a consequence, mitochondrial dysfunction manifests commonly as multisystem disorders, with frequent involvement of high energy demand tissues, such as the brain, skeletal muscle, and heart [3]. The OxPhos system consists of 85 protein subunits, and is under the control of two genomes, the nuclear DNA (nDNA) and mitochondrial DNA (mtDNA). Mitochondrial diseases (MtDs) can be due to pathogenic variants in either [4]. Due to their genetic complexity and the variegated biochemical functions of mitochondria, MtDs are clinically and molecularly heterogeneous and present diagnostic, clinical management, and therapeutic challenges. Although MtDs are rare diseases (< 5 per 10,000 persons in Europe, and < 200,000 individuals in the United States [5], among rare disorders they are relatively frequent, with an overall estimated prevalence of 11.5:100,000 [4]. In aggregate, MtDs represent the most prevalent group of inherited neurological disorders [6].

**Table 1:** Glossary of key definitions

ATP: Adenosine triphosphate
MtDs: Mitochondrial disorders
NDOCS: Number of Doctors Seen during the diagnostic process
NAMDC: The North American Mitochondrial Disease Consortium
OD1: 2015–2016 Odyssey1 survey of patients with a medical diagnosis of mitochondrial disease
OD2: 2018 Odyssey2 survey of patients with a medical diagnosis of mitochondrial disease
PCP: Primary Care Physician
RDCRN: The Rare Disease Clinical Research Network
TOD: Time from symptom Onset to mitochondrial disease Diagnosis
WES: Whole exome sequencing

An initial Odyssey1 (OD1) survey analyzed data collected in 2016 from 210 Rare Disease Clinical Research Network (RDCRN) Contact Registry and North American Mitochondrial Mitochondrial Disease Consortium (NAMDC) Registry participants who were patients with a biochemical deficiency or self-reported diagnosis of mitochondrial disease, or their caregivers [7]. The survey showed that patients consulted many clinicians (mean > 8, median 5), the first typically (56.7%) a primary care physician (PCP), although one-third (35.2%) initially sought a specialist. 55.2% received their diagnosis from a neurologist, 18.2% from a clinical geneticist, and 11.8% from a metabolic disease specialist. A majority (54.6%) received one or more non-mitochondrial diagnoses before their final mitochondrial diagnosis. The OD1 survey thus showed that the diagnostic odyssey of MtD patients is complex and burdensome. It features multiple consultations and tests, and frequent conflicting diagnoses, reflecting disease variety, prolonged diagnostic uncertainty, and clinician unfamiliarity. The original survey provided a significant benchmark: we called for its replication at appropriate intervals.

The current paper uses data from the 2018 NIH-funded Odyssey 2 (OD2) survey (N = 215) to replicate OD1 survey questions and address others on which we lack reliable knowledge. We focus primarily on the Time from Onset of first symptoms to Mitochondrial Diagnosis (TOD); the Number of Doctors patients see before mitochondrial diagnosis (NDOCS); and what factors may reduce or extend TOD and NDOCS. We devote substantial efforts to obtaining the highly complete data across the multiple stages of the diagnostic odyssey that are essential to address these questions, but generally unavailable.

## Materials and methods

The OD2 survey target population, as in the OD1 survey, was patients with a medical diagnosis of mitochondrial disease, i.e., who: (1) reported receiving a diagnosis of mitochondrial disease from a doctor; (2) were able to provide informed consent; and (3) were capable of completing the survey. The questionnaire (Diagnostic Odyssey Survey 2, see Additional file 3) was administered to both members of the RDCRN NAMDC Contact Registry, which provided all of the patients for the OD1 survey [8]; and the Mitochondrial Disease Community Registry (MDCR), maintained by the United Mitochondrial Disease Foundation (UMDF), a leading patient representative group [9].

The OD2 survey, administered September–December 2018, was sent simultaneously to 1265 RDCRN-NAMDC and 963 UMDF-MDCR registrants with email addresses. Respondents who received two survey copies were instructed to complete only one. 253 individuals responded, of whom 215 were eligible for analysis (i.e., answered “No” to “I have previously completed the Odyssey2 Survey”, and “Yes” to “Have you been informed by a doctor that you have mitochondrial disease?”). To maximize analyzable data, three clinicians (AK, MH, and DD) independently recoded responses to text and “other” responses to analyzable categories. AK and MH independently coded all responses, and DD was the independent tiebreaker when needed. Consensus was achieved on all recodes.

### Statistical analysis

We report major response distributions, and the results of four comparisons: (i) all answers on key questions between and within RDCRN and MDCR respondents, to establish if any individuals completed the survey twice; (ii) results before and after coding text responses, to assess whether this meaningfully increased the number of analyzable responses or qualitatively changed the clinical picture; (iii) RDCRN and MDCR respondents’ answers, to establish if it was appropriate to combine them for analysis; and (iv) TOD and NDOCS in patients who saw a PCP and those who saw a specialist for their first symptoms.

Frequency counts and percentages were compared with t-tests, Fisher’s exact tests for proportions, Chi-squared tests, and Wilcoxon rank-sum and Kruskal–Wallis tests for medians, using R version 4.0.2 (R Foundation for Statistical computing). All results were confirmed using SAS version 9.4 (SAS Institute, Cary, North Carolina). A Sankey plot and other figures provide data visualization.

## Results

**Fig. 1:**
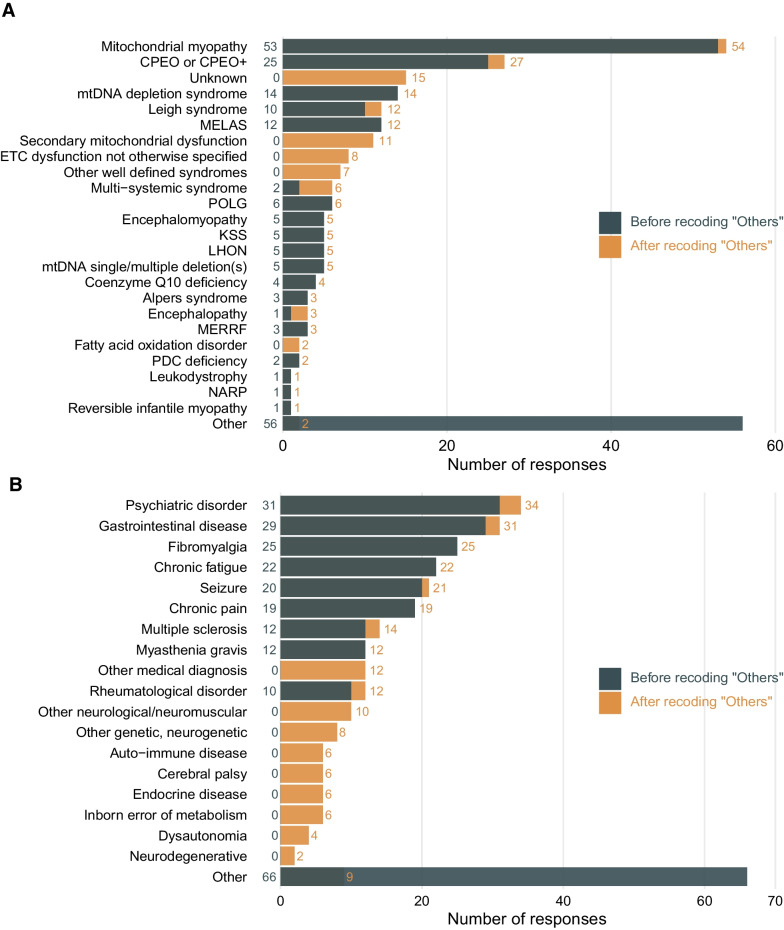
**A** Self-reported mitochondrial disease diagnoses before and after expert recoding of text responses. Each patient provides 1 response. N = 215. POLG, Polymerase gamma related disorders/ataxia neuropathy spectrum; KSS, Kearns–Sayre syndrome; LHON, Leber hereditary optic neuropathy; MERRF, Myoclonic epilepsy with ragged-red fibers; PDC, Pyruvate dehydrogenase complex; NARP, Neuropathy, ataxia, and retinitis pigmentosa. **B** Self-reported prior non-mitochondrial disease diagnoses before and after expert recoding of text responses each patient can provide more than one response. "Other" responses could be recoded into several existing or new responses. N = 259 responses after recoding, 246 responses before

Of the 215 eligible respondents, 122 (56.7%) are from the NAMDC and 93 (43.3%) from the MDCR registries. Importantly, there were no duplicate respondents across or within the two registries (see Additional file 1: Table S1). Coding text responses increased analyzable observations by 22.7% for type of specialist with whom patients first discussed symptoms; 38.9% for prior non-mitochondrial diagnosis; and 34.2% for mitochondrial diagnosis (see Additional file 1: Table S2). Figure 1 shows the distributions of both of these diagnoses, before and after recoding. Since data from the two registries did not differ statistically on current or prior diagnosis, age, or other key questions (see Additional file 1: Table S3), they were combined for analysis.

**Fig. 2:**
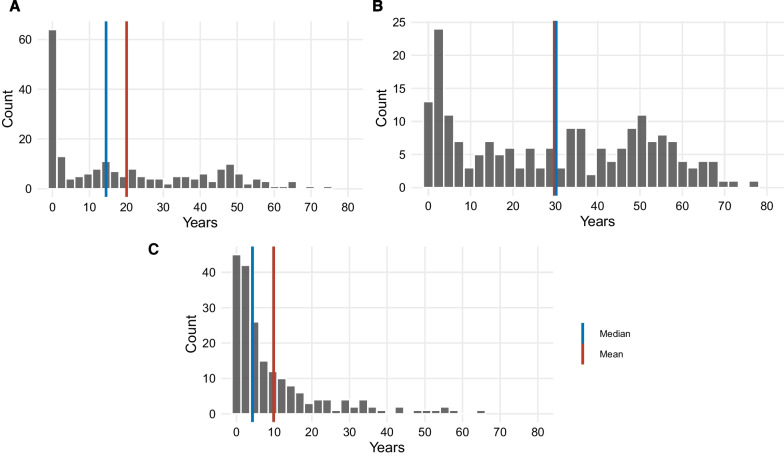
**A** Age at first symptom(s) of mitochondrial disease (N = 209); **B** Age at diagnosis of mitochondrial disease (N = 198); **C** Time from first symptoms to diagnosis of mitochondrial disease (N = 198)

Median age at first symptoms was 14.5 years, median age at diagnosis 30.2, and median TOD 4.2 years, with mean 9.9, reflecting outliers with high values (Table 2 and Fig. 2). TOD ranged from 12.8 to 65.4 years in the upper quartile and 0 (rounded) to 1.5 years in the lower. Median TOD was 2.9 years for early onset patients (< 2 years old), and 5.8 years for older patients (*p* = 0.07). TOD did not differ in patients with classic and non-classic clinical syndromes (4.0 vs 4.1 years, *p* = 0.24, Table 2). For the 170 patients with all three measurements, mean time from first visit with a doctor to diagnosis (7.0 years) was longer than mean time from first symptoms to first visit with a doctor (3.6 years). Additional file 2: Figure S1 shows these data for each patient.

**Table 2:** Medians and means, in years for: age at onset of first symptoms for mitochondrial disease, age at diagnosis of mitochondrial disease, and time from onset of first symptoms to diagnosis (TOD) (panel A); TOD by dichotomized age at symptom onset (panel B); TOD by trichotomized age at symptom onset (panel C); and TOD by clinical syndrome of first symptoms (panel D)

	N	Median	Interquartile Range	Mean (SD)
A *Medians and means for age at onset of first symptoms of mitochondrial disease, age at diagnosis, and TOD*				
Age at onset	209	14.5	37.8	20.1 (20.3)
Age at diagnosis	198	30.2	43.1	29.7 (22.0)
Time from onset to diagnosis^a^	198	4.2	11.3	9.9 (13.0)
B *TOD by dichotomized age at symptom onset* (N = 198)				*p* = 0.07^b^
< 2	68	2.9	8.1	9.4 (15.0)
≥ 2	130	5.8	11.6	10.2 (11.6)
C *TOD by trichotomized age at onset* (N = 198)				*p* = 0.09^c^
< 2	68	2.9	8.1	9.4 (15.0)
2–< 12	23	5.3	23.8	15.8 (17.6)
≥ 12	107	5.8	10.6	8.9 (9.9)
D *TOD by clinical syndrome of first symptoms* (N = 182)^d^				*p* = 0.24^b^
Classic^e^	65	4.0	10.6	8.3 (11.2)
Non-classic	117	4.1	11.3	9.9 (13.1)

^a^17 patients missing year of diagnosis excluded. Missing months were recoded to 6

^b^Wilcoxon rank-sum test

^c^Kruskal–Wallis test

^d^16 patients with unknown syndromes excluded

^e^Classic syndromes include (1) CPEO or CPEO-plus (2) MELAS (3) Leigh syndrome (4) LHON (5) Kearns-Sayre syndrome (6) MERRF (7) NARP (8) Alpers-Huttenlocher syndrome (Alpers syndrome) (9) MNGIE (10) Reversible infantile myopathy with cytochrome c oxidase deficiency (11) Aminoglycoside-induced deafness (12) Barth syndrome (13) Maternally Inherited Diabetes and deafness (MIDD) (14) Pearson syndrome. Non-classic syndromes include (1) multisystemic syndrome (2) encephalomyopathy (3) Mitochondrial myopathy (4) Mitochondrial DNA (mtDNA) depletion syndrome (5) Secondary mitochondrial dysfunction (primary genetic condition not mitochondrial) (6) ETC dysfunction not otherwise specified (7) Other well defined syndrome (8) Polymerase gamma (POLG) related disorders/ataxia neuropathy spectrum (9) Mitochondrial DNA (mtDNA) deletion(s) (10) Coenzyme Q10 deficiency (11) Encephalopathy (12) Fatty acid oxidation disorder (13) Pyruvate dehydrogenase deficiency (PDH) (14) Leukodystrophy (15) Hepatocerebral syndrome (16) Pyruvate carboxylase deficiency (PC) [1]

Patients saw a mean of 6.7 ± 5.2 doctors, median 5 (see Additional file 1: Table S4 and Additional file 2: Figure S2). The range was 10–20+ in the upper quartile, and 1–3 in the lower. Although patients who first saw a specialist saw fewer doctors before their final diagnosis than those who first saw a PCP (5.2 vs 7.8, *p* =  < 0.001), their overall TOD was non-significantly longer (10.4 vs 9.4 years, *p* = 0.61), rather than shorter.

More than 27 distinct clinical manifestations prompted patients to consult a physician: over half of the patients (125/215, 58.1%) reported more than one. Fatigue (13.8%) was the most frequent motivation, followed by weakness (12.8%), difficulty walking (8.5%), and GI dysfunction (8.1%): see Additional file 1: Table S5.

**Fig. 3:**
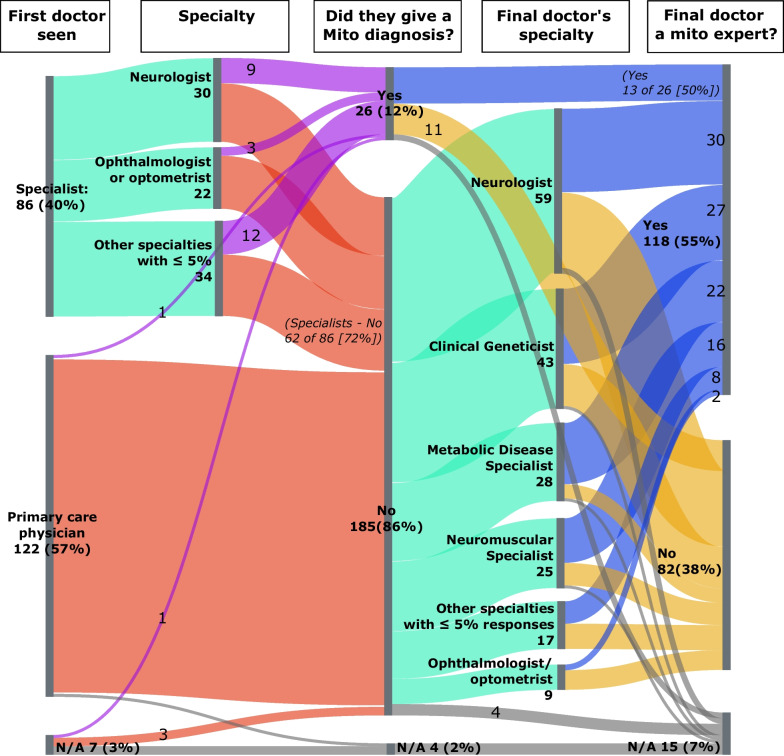
Evolution of mitochondrial disease diagnosis by specialty of diagnosing clinician. At each node (First Doctor Seen; Specialty; Did they give a mito diagnosis?; Final doctor’s specialty; and Final Doctor a Mito Expert?), Ns (%s) sum vertically. (N = 215 patients)

Figure 3, a Sankey plot, summarizes the evolution of the diagnostic odyssey, presenting the process in stages. It covers first doctor seen, their specialty, who gave a mito diagnosis then, and final doctors specialty, etc. It shows the many initial misdiagnoses, the corrections made as the process continues, and the specialties of the clinicians who made them (see also Additional file 1: Table S6).

Half of the patients (104 of the 204 responding, 51.0%) had received a prior non-mitochondrial disease diagnosis (see Additional file 1: Table S7). The most frequent reason for seeking a further diagnosis was that symptoms had not improved with treatment (64 of 223 responses [28.7%]).

After receiving their mitochondrial diagnosis, 72.3% of responders (149 of 206) experienced 441 disease management or treatment changes. Most (86%) recorded more than 1 change, with a mean of 3 (Additional file 1: Table S8 panel 1). These changes not only pertained to an actionable therapeutic intervention (medication, diet, exercise therapy change: see Additional file 1: Table S8) but also to a subjective change in the healthcare provider’s perception of the patient’s experience (Table 2). Sixty-five (32.2%) of the 202 patients who provided details on changes in disease management or treatment after receiving genetic confirmation of their diagnosis recorded 184 changes (mean 2.9 ± 1.4). Of those, 78.5% (51) reported more than one change (Additional file 1: Table S8 panel 2). While more than 70% of patients experienced a change in treatment management after diagnosis overall, only one-third experienced such a change after genetic confirmation. The 98 participants (47.6% of 206) with health care team changes recorded 151 new referrals (see Additional file 1: Table S9).

About half (100 of 203, 49.3%) of the respondents reported a change of perception among health care professionals after diagnosis (Table 3). Most described beneficial outcomes after mitochondrial disease diagnosis, perceiving that their care providers took their symptoms more seriously (18.7%), gave up a mental health diagnosis as the cause of their symptoms (12%), and increased empathy towards them (6%), which ultimately led to a change in management (8.7%). A small minority of patients experienced a negative impact from receiving their diagnosis: 2% stated that their doctor was skeptical of the diagnosis or took symptoms less seriously (0.7%). 6% reported that after a diagnosis of mitochondrial disease, their treating doctor displayed lack of knowledge or experience in the matter, and in some cases became dismissive of their care.

**Table 3:** Changes reported by patients in health professionals’ perception of them after their mitochondrial diagnosis. N = 150 responses from 203 patients

Changes	N	%
Took symptoms more seriously	28	18.7
Now had cause(s) and explanations for symptoms	21	14.0
Gave up mental health diagnoses or psychological or psychosomatic interpretations	18	12.0
Changed management of symptoms, or treatment decisions, or interventions	13	8.7
Increased understanding for symptoms/disease	10	6.7
Increased empathy/respect for patient	9	6.0
Displayed lack of knowledge of or experience with mitochondrial diseases	6	4.0
Prompted education/reading about mitochondrial diseases	5	3.3
Were skeptical of diagnosis	3	2.0
Displayed lack of knowledge of or experience with mitochondrial diseases, plus dismissive attitude	3	2.0
Gave up alternative diagnoses or potential diagnoses	3	2.0
Looked for other options for treatment	2	1.3
Concluded condition was fatal	2	1.3
Led to referral to mitochondrial expert/mitochondrial disease center	2	1.3
Ended diagnostic testing	2	1.3
Reduced rerunning of invasive tests	1	0.7
Took symptoms less seriously	1	0.7
Instituted treatment that improved symptoms or quality of life	1	0.7
Led to additional testing	1	0.7
Ended suspicions of child abuse	1	0.7
Other	2	1.3
Unknown	16	10.7
Total	150	100

100 (49.3%) of the 203 patients reported a mean of 1.4 ± 0.9 changes. 33 (33.3%) reported more than 1. 87 patients reported no change. 12 patients who did not respond are excluded

One hundred fifty-one patients (74.0%) reported joining support and advocacy groups (281 new memberships; mean 1.9 ± 1.0 per patient). 80 (53.0%) recorded more than one type of group (Additional file 1: Table S10). 122 (81.3%) considered joining communities beneficial, and 108 of them reported details of 188 specific benefits in total, with a mean of 1.7 ± 0.9. Fifty-seven (46.7%) reported more than one benefit (see Additional file 1: Table S11). Most patients anticipated an impact if their diagnosis proved wrong: 90 (45%) expected that it would be negative, and 53 (26.5%) that it would be positive (see Additional file 1: Table S12).

## Discussion

### The importance of complete data, collected consistently across surveys

Reliably characterizing the evolution of a multistep process such as the mitochondrial diagnostic odyssey, and the factors which impact it, requires requires data that are representative, and highly complete across all stages.

A key OD2 survey design concern was that diagnosed patients may be demotivated from survey participation, and therefore less likely to respond to surveys, because of several factors such as long TOD, survey complexity, multiple survey requests, and concerns about data confidentiality and inappropriate future use. To minimize this potentially very important selection bias, the OD2 survey, like OD1, employed the simplest possible (IRB-approved) anonymized enrollment, and short survey length. We consider these major design strengths.

In addition, recoding “other” responses where adequate detail was provided increased analyzable data on both prior non-mitochondrial and final mitochondrial diagnoses by over one-third. Given this, and the fact that the diagnostic odyssey deals with intense personal experiences, which can be expected to change going forward, we recommend continuing this manual recoding effort as needed in future work. Experienced clinicians are recommended as coders. Validation procedures, such as our use of a team of three clinical experts, ensure reliability. Maintaining these procedures will improve our characterization of the progression from pre-mitochondrial to mitochondrial diagnosis, and our ability to address other major questions.

Question wording in the OD2 survey was also kept as consistent as possible with OD1.

### Specific conclusions

The OD2 survey responses are generally similar to those from OD1, but more recent and extensive, and provide more analyzable data. They increase our knowledge of the challenges and burden faced by patients, and in some ways show them to be more severe than previously reported, particularly by providing, to the best of our knowledge, the first extensive data on TOD. The longer the TOD, the greater the risk of iatrogenic complications from unnecessary doctor visits, tests and therapies—an “adverse multiplier” effect. Given the great TOD length and clinical burden, and the many clinicians consulted, there is substantial potential for shortening the odyssey. More prompt patient contact with primary mitochondrial disease specialists, and early implementation of appropriate tests, may enable improvement—but only so long as the procedures do not themselves generate offsetting further testing or investigations.

#### Time from disease onset to diagnosis

Mean TOD (9.9 ± 13.0 years) is high, and substantially greater than the 4–5 years reported for rare diseases generally [10]. Given the wide TOD variability (Additional file 1: Table S4), results require disaggregation. While a 6-month TOD reduction in the upper TOD quartile would have little clinical importance, in the lowest quartile it would be of a great benefit, particularly for those with symptom onset by age 2. As a corollary, for the upper quartile of patients, with their TOD range of 13–65 years, a mitochondrial diagnosis within 2–3, or even 5, years of symptom onset would dramatically reduce their burden, if it is accompanied by efficient testing and treatment. In assessing progress, reporting improvement in TOD and NDOCS by quartile (or quintile or decile) is therefore recommended, to avoid unduly optimistic or pessimistic conclusions. The usual practice of reporting these results by dichotomy (sometimes trichotomy), does not seem optimal, given the variability of these outcomes.

Our analysis relies on patient reports of time of symptom onset and diagnosis by a clinician. An ideal analysis would be based directly on clinical records, but these are not yet available. (See the comments on EHRs below.) We are participating actively in efforts at improvement. But given no current better alternative(s), our results provide the best available benchmarks. One important question is whether the key findings, such as the length of TOD, are likely to be biased towards over- or under-estimation. While TOD in the upper quartile is long, mitochondrial disease diagnosis is notoriously difficult, and our results indicate that even among those eventually diagnosed, almost none are diagnosed when their first contact is a PCP. Given this, it is possible that the true extent of TOD is at least as high as we find in the OD2 survey.

#### Number of doctors seen during the diagnostic process

Mean NDOCS of 6.7 ± 5.2 is high. While first meeting a specialist rather than a PCP significantly reduced it (from 7.8 for first meeting a PCP to 5.2), it did not reduce TOD, which increased (insignificantly) by a year (see Results). The reasons for this apparent paradox are unknown. They require exploration, and data which we currently lack. One possible explanation is the extended wait time to see specialists. This result cautions against the assumption of lockstep correlation among outcomes, and undue optimistic generalization based on only one of them. It also raises the possibility that some steps initially expected to shorten the odyssey may actually lengthen it. Research to identify which steps actually bring the hoped-for benefits, and which do not, is vital.

#### Current mitochondrial disease diagnostic strategies

Although the majority of patients (55%) were diagnosed by a mitochondrial disease specialist, a substantial proportion (38%) of final diagnoses were provided by specialists who were not mitochondrial experts. This suggests that relying on non-mitochondrial specialists can be a path forward, but what specific actions by them shorten or extend the odyssey are currently opaque.

Additional file 1: Table S5 confirms and extends previous findings on the complexity of mitochondrial disorders [11]. The 494 initial manifestations from the 215 patients, spanning more than 25 categories, highlight the multisystemic nature of mitochondrial diseases, and likely contribute to the high proportion of prior non-mitochondrial diagnoses (51% of respondents [Additional file 1: Table S7], similar to the 54.6% in the OD1 survey). The data also document the substantial impact of mitochondrial diagnosis. Patients report substantial treatment changes, largely but not invariably seen as beneficial, and increased membership and strong support of advocacy groups (see Additional file 1: Tables S8 and S9). Given these results, the great challenge is to reduce TOD and the number of doctors who patients consult, so that they can more rapidly receive the substantial, confirmed benefits of diagnosis.

The OD2 survey design excludes patients with mitochondrial disease who embarked on the odyssey but were not successfully diagnosed. Including them in a time-to event analysis may increase estimated mean TOD. Establishing what factors increase the probability of these diagnostic failures may prove highly beneficial and is recommended. The limitations of patient reports also precluded collecting data on the impact on TOD of unbiased diagnostic tests such as mitochondrial DNA sequencing, sequencing of nuclear gene(s), and WES. Their increased use is widely recommended as a first-tier diagnostic tool in patients with suspected mitochondrial disorder. This may shorten TOD, but confirmation is needed [12]. Again, the hard challenge is to find which tests are more and which tests are less beneficial, and for which subgroups. Our perspective allows for and encourages searching for a range of diagnostic trajectories which may differ across subgroups. Our methods must allow for all outcome possibilities. Given our abundant knowledge of the great complexity of mitochondrial disorders, it is reasonable to expect multiple response patterns combining successes and (correctable) failures.

In principle, EHRs offer the potential to provide comprehensive data to detect and diagnose rare diseases, to characterize more fully the true extent and details of the mitochondrial diagnostic odyssey, and to shorten it going forward [13]. While they have appeal and their use is strongly encouraged, the challenges of showing their validity, reliability, and clinical utility are as yet unmet [14]. They also do not provide the direct and indispensable patient voice that is available from surveys such as OD2. As usual in complex situations, no single strategy provides all the answers. Both should be pursued going forward: reconciling them, to the extent that this is possible, is an important challenge.

### The path forward: a two-pronged approach

In overview, we recommend a strategy with two main elements. The first is the use, as early as possible, of advanced diagnostic techniques such as gene panels and WES. Although these are indispensable [7, 15–19], we have not investigated them here only because of the limitations of patient reports.

Although use of gene panels and WES is increasing, it is subject to major limitations which cannot realistically be expected to disappear in the immediate or medium term. These bottlenecks include the small number of specialist mitochondrial clinicians; the resulting length of time to obtain referrals, access and appointments to them; and the fact that work-ups by medical geneticists often include very long delays between appointments. Partly because of those factors, and partly because of others revealed by our results (for example, physician attitudes to undiagnosed patients show that patients with undiagnosed mitochondrial diseases suffer greatly from their doctors’ unfamiliarity with these diseases), the second approach is to promote continuing education of physicians who are not MtD specialists. Medical training and continuing education can promote awareness that molecular work-up to screen for MtD and other genetic disorders should be employed earlier in the evaluation of patients who are difficult to diagnose or do not respond to treatment. CE measures can also include toolkits such as up-to-date algorithmic approaches to molecular diagnostics. The need is to combine the two approaches rather than to prioritize one over the other.

Building upon our findings, more detailed work is needed to assess the relative impact of support from PCPs and mitochondrial specialists, and above all to identify which specific combinations of sequential steps drawn from both can best reduce the overall diagnostic odyssey. Needed elements include accurate diagnoses; complete data on these and other key variables on all patients at every key odyssey stage, involving all consulted clinicians; and importantly, the application of appropriate statistical techniques to model the complex multicomponent sequences involved. Combining all of these is our challenge going forward. This comprehensive strategy may be somewhat reassuring to patients and patient representative groups, and may help to allay their reported concerns. It may also be relevant to other rare diseases where similar patterns apply, such as for example in genetic neuromuscular diseases, congenital disorders of glycosylation, and genetic causes of developmental delay [20–22].

## Limitations

Given the anonymized design of the OD1 and OD2 surveys, respondents common to both cannot be identified. The two surveys therefore do not provide estimates of changes over time.

The complexity imposed by not collecting the OD2 survey data in a single initial database delayed analysis because of the need to recode all responses in a single consistent scheme. This approach should be avoided.

## Supplementary Information

**Additional file 1: Table S1**. Discrepant responses for individuals between and within registries on 15 major variables. N = 11,346 comparisons. **Table S2**. Number of analyzable responsesbefore and after expert coding of text responses, and % increase after coding, for questions allowing 1 response per patient, and questions allowing more than 1 response per patient. From 215 patients. **Table S3**. Comparisons of key results in the NAMDC and UMDF registries, with p-values for differences, for binaryoutcomes; categorical variables with 3 or more categories; and quantitative variables. **Table S4**. Quartile values for Time in years from symptom Onset to mitochondrial disease Diagnosisand Number of Doctors Seen during the diagnostic process. **Table S5**. Distribution of symptomsmotivating initial consultation with a doctor. N = 494 responses from 215 patients. **Table S6**. Distribution of specialtiesof physicians who provided the mitochondrial diagnoses. N = 205 patients. **Table S7**. Distribution of motivationsfor seeking a further diagnosis, from patients with a prior non-mitochondrial diagnosis. N = 223 responses from 104 patients. **Table S8**. Changesin disease management or treatment after mitochondrial disease diagnosis, Panel A; and after genetic confirmation of mitochondrial disease diagnosis, Panel B. **Table S9**. Changesin health care team after a mitochondrial disease diagnosis. N = 151 responses from 98 patients. **Table S10**. Groupsjoined after mitochondrial disease diagnosis. N = 281 responses from 151 patients. **Table S11**. Benefitsof joining groups. N = 188 responses from 122 patients. **Table S12**. Anticipated impact if mitochondrial diagnosis is wrong. N = 200 patients.

**Additional file 2: Figure S1.** Individual patient trajectories, from age in years at first symptoms of mitochondrial disease, through age at first visit to doctor, to age at diagnosis, sorted by age at first visit to doctor. 45 cases with missing or contradictory information are omitted.. **Figure S2**. Distribution of Number of Doctors Seenby patients with mitochondrial disease from symptom onset to diagnosis..

**Additional file 3**. The Diagnostic Odyssey 2 Survey Questionnaire.

## Data Availability

The data underlying the results presented in the study are available from J.LP. Thompson, (jlt12@cumc.columbia.edu).
