# Correction to: The evolution of the mitochondrial disease diagnostic odyssey

**DOI:** 10.1186/s13023-023-02832-0

**Published:** 2023-07-20

**Authors:** John L. P. Thompson, Amel Karaa, Hung Pham, Philip Yeske, Jeffrey Krischer, Yi Xiao, Yuelin Long, Amanda Kramer, David Dimmock, Amy Holbert, Cliff Gorski, Kristin M. Engelstad, Richard Buchsbaum, Xiomara Q. Rosales, Michio Hirano

**Affiliations:** 1 Department of Biostatistics, Mailman School of Public Health, Columbia University Irving Medical Center, New York, USA; 2 Division of Genetics, Massachusetts General Hospital/Harvard Medical School, Boston, MA USA; 3 United Mitochondrial Disease Foundation, Pittsburgh, Pennsylvania USA; 4 University of South Florida Health Informatics Institute, Tampa, FL USA; 5 Department of Population and Family Health, Mailman School of Public Health, Columbia University, New York, USA; 6 Creyon Bio, San Diego, CA USA; 7 Tampa General Hospital, Tampa, FL USA; 8 Department of Neurology, Columbia University Irving Medical Center, New York, USA

**Correction to: Orphanet Journal of Rare Diseases**

10.1186/s13023-023-02754-x

Following publication of the original article [1], we have been notified that affiliation 3 was published incorrectly. It should be as follows:

3 United Mitochondrial Disease Foundation, Pittsburgh, Pennsylvania, United States of America.

The original article has been corrected.
