# Supplementary material for: The evolution of the mitochondrial disease diagnostic odyssey
Source: Orphanet J Rare Dis. 2023 Jun 22;18:157. doi: 10.1186/s13023-023-02754-x (PMC10288668; doi:10.1186/s13023-023-02754-x)
Supplement: Supplementary file 1 — Additional file 1: Table S1. Discrepant responses for individuals between and within registries on 15 major variables. N = 11,346 comparisons. Table S2. Number of analyzable responsesbefore and after expert coding of text responses, and % increase after coding, for questions allowing 1 response per patient, and questions allowing more than 1 response per patient. From 215 patients. Table S3. Comparisons of key results in the NAMDC and UMDF registries, with p-values for differences, for binaryoutcomes; categorical variables with 3 or more categories; and quantitative variables. Table S4. Quartile values for Time in years from symptom Onset to mitochondrial disease Diagnosisand Number of Doctors Seen during the diagnostic process. Table S5. Distribution of symptomsmotivating initial consultation with a doctor. N = 494 responses from 215 patients. Table S6. Distribution of specialtiesof physicians who provided the mitochondrial diagnoses. N = 205 patients. Table S7. Distribution of motivationsfor seeking a further diagnosis, from patients with a prior non-mitochondrial diagnosis. N = 223 responses from 104 patients. Table S8. Changesin disease management or treatment after mitochondrial disease diagnosis, Panel A; and after genetic confirmation of mitochondrial disease diagnosis, Panel B. Table S9. Changesin health care team after a mitochondrial disease diagnosis. N = 151 responses from 98 patients. Table S10. Groupsjoined after mitochondrial disease diagnosis. N = 281 responses from 151 patients. Table S11. Benefitsof joining groups. N = 188 responses from 122 patients. Table S12. Anticipated impact if mitochondrial diagnosis is wrong. N = 200 patients. [file 13023_2023_2754_MOESM1_ESM.pdf]

S1 Table. Discrepant responses for individuals between and within registries on 15 major variables. N = 11,346 comparisons.

Groups/N of pairs[a]	Discrepancies			
	Minimum	Mean (SD)	Median (IQR)	Maximum
RDCRN and MDCR/11,346	5	11.3 (1.7)	12.0 (2.0)	15
Within RDCRN/7,381	6	11.4 (1.5)	12.0 (2.0)	15
Within MDCR/4,278	2	11.2 (1.9)	11.1 (3.0)	15

[a]We compared 1) the responses for every RDCRN patient to those for every MDCR patient, and 2) within each registry, the responses for every patient, on each of 15 major variables. 15 discrepancies, the maximum possible, indicates not a duplicate: no discrepancies, or a small number, suggests a possible duplicate. The discrepancies were tightly grouped around a high median of 11 or 12. In addition, our clinical experts examined the responses between the registries, the main concern, for the patients with the 7 lowest numbers of discrepancies and concluded that none of them were duplicates.

S2 Table. Number of analyzable responses (all responses except Other or missing) before and after expert coding of text responses, and % increase after coding, for questions allowing 1 response per patient (Panel A), and questions allowing more than 1 response per patient (Panel B). From 215 patients.

	N before/after coding	% increase
**A Questions allowing 1 response per patient**		
Confirmed mitochondrial disease diagnosis	158/212	34.2
Specialty of doctor with whom a patient first discussed symptoms	66/81	22.7
Specialty of doctor who diagnosed mitochondrial disease	160/176	10.0
How did disease management change after receiving genetic confirmation for mitochondrial disease diagnosis	171/184	7.6
**B Questions allowing more than 1 response per patient**		
Prior non-mitochondrial diagnoses	180/250	38.9
Specialty of health care professionals on care team before receiving mitochondrial disease diagnosis	402/466	15.9
Motivation to seek different diagnosis after receiving non-mitochondrial disease diagnosis	195/222	13.9
Symptoms motivating patients to see doctor for 1st time	420/471	12.1
Biochemical deficiency or biochemical diagnosis	74/82	10.8
Symptoms attributed to earliest onset of mitochondrial disease	502/547	9.0
How did disease management change after receiving mitochondrial disease diagnosis	415/441	6.3
**Total**	**2743/3132**	**14.2**

S3 Table. Comparisons of key results in the NAMDC and UMDF registries, with p-values for differences, for binary (yes/no) outcomes (Panel A); categorical variables with 3 or more categories (Panel B); and quantitative variables (Panel C).

	NAMDC	UMDF		TOTAL
**A Binary (yes/no) outcomes**	yes/total, (%)	yes/total (%)	p[a]	yes/total (%)
Prior non-mitochondrial diagnoses	56/119 (47.1)	48/85 (56.5)	0.20	104/204 (51.0)
Confirmed biochemical deficiency	22/115 (19.1)	16/90 (17.8)	0.86	38/205 (18.5)
Mitochondrial disease diagnosis from a mitochondrial disease expert	76/117 (65.0)	42/83 (50.6)	0.06	118/200 (59.0)
Disease management changed after mitochondrial diagnosis	85/121 (70.2)	64/85 (75.3)	0.53	149/206 (72.3)
Health care team changed after mitochondrial disease diagnosis	55/121 (45.5)	43/85 (50.6)	0.48	98/206 (47.6)
Health professionals' perception changes, in patients' view	56/119 (47.1)	44/84 (52.4)	0.46	100/203 (49.3)
Joined a group after mitochondrial disease diagnosis	88/122 (72.1)	63/82 (76.8)	0.52	151/204 (74.0)
Consider joining a group beneficial	73/88 (83.0)	49/62 (79.0)	0.67	122/150 (81.3)

[a] Fisher's exact test.

	NAMDC	UMDF		TOTAL
**B Categorical variables with 3 or more categories**	N	N	P[b]	N
Self-reported mitochondrial disease	121	93	0.31	214
Specialty of doctor who diagnosed the mitochondrial disease	121	84	0.58	205
Impact of wrong diagnosis	119	81	0.49	200
Disease management changed as result of genetic confirmation	117	85	0.11	202

[b]Chi-Squared test.

	NAMDC		UMDF			TOTAL	
**C Quantitative variables**	N	Med/IQR<br>Mean/SD	N	Med/IQR<br>Mean/SD	P[c]	N	Med/IQR<br>Mean/SD
Time to diagnosis	114	4.0/11.4<br>8.9/11.6	84	4.7/11.4<br>11.2/14.7	0.29	198	4.2/11.3<br>9.9/13
Age at questionnaire	120	45.1/40.3<br>39.2/22.2	92	40.6/42.9<br>39.2/23.4	0.90	212	42.7/41.1<br>39.2/22.7
Age at onset	119	15.5/37.5<br>20.4/20.0	90	13.3/34.4<br>19.6/20.8	0.60	209	14.5/37.75<br>20.1/20.3
Age at diagnosis	114	32.8/41.5<br>29.0/21.2	84	29.6/44.6<br>30.8/23.1	0.51	198	30.2/43.1<br>29.7/22.0

[c]Wilcoxon rank-sum test for medians

S4 Table. Quartile values for Time in years from symptom Onset to mitochondrial disease Diagnosis (TOD) and Number of Doctors Seen during the diagnostic process (NDOCS)

	TOD N=198	NDOCS N=204
100% Max	65.38	20+*
75% Q3	12.84	10
50% Median	4.23	5
25% Q1	1.50	3
0% Min	0	1

*The highest response category was 20 or more.

S5 Table. Distribution of symptoms (N, %) motivating initial consultation with a doctor. N=494 responses from 215 patients.[a]

Symptom	N	%
Fatigue	68	13.8
Weakness	63	12.8
Difficulty walking	42	8.5
Gastrointestinal discomfort or dysfunction	40	8.1
Developmental delay	35	7.1
Floppiness/hypotonia	30	6.1
Droopy eyelids	26	5.3
Failure to grow or gain weight	25	5.1
Numbness, weakness in hands and/or feet	24	4.9
Impaired coordination	19	3.8
Seizures	18	3.6
Loss of vision	15	3.0
Change in mental health	11	2.2
Ophthalmoplegia	9	1.8
Hearing loss	8	1.6
Headaches/Migraines	7	1.4
Muscle disease	7	1.4
Pain	6	1.2
Diabetes	4	0.8
Heart disease	4	0.8
Shortness of breath	3	0.6
Diagnosed due a known family history	2	0.4
Unknown	2	0.4
Kidney disease	1	0.2
Liver disease	1	0.2
Exercise intolerance	1	0.2
Other	23	4.7
**Total**	**494**	**100**

[a]213 (99%) of 215 patients reported a mean of 2.3±1.7 motivating symptoms. 125 (58.1%) reported more than 1. 88 reported 1. Two patients who did not respond are excluded.

S6 Table. Distribution of specialties (N, %) of physicians who provided the mitochondrial diagnoses. N=205 patients.[a]

Specialty	N	%
Neurologist or Neuromuscular Specialist	94	45.9
Clinical Geneticist	46	22.4
Metabolic Disease Specialist	31	15.1
Ophthalmologist/optometrist	12	5.9
Rheumatologist	8	3.9
Endocrinologist	2	1.0
Infectious Disease	2	1.0
Pulmonologist	2	1.0
Unknown	2	1.0
PCP, Internist, Family Medicine or pediatrician	1	0.5
Other	5	2.4
**Total**	**205**	**100**

[a]181 specialties were provided in response to question 12, and 24 in response to question 10 in the Odyssey 2 survey, which is included as an Appendix in the Supplementary material. 10 patients who did not respond are excluded.

S7 Table. Distribution of motivations (N, %) for seeking a further diagnosis, from patients with a prior non-mitochondrial diagnosis. N=223 responses from 104 patients.[a]

Motivation	N	%
My symptoms were not improving with treatment.	64	28.7
Consultation(s) with other doctors or health care professionals	61	27.4
Web search or browsing	31	13.9
Reading magazine(s), journal, or other print media	16	7.2
Consultation with people not in the medical profession	14	6.3
Information from a patient group.	9	4.0
My symptoms/signs did not quite fit the diagnosis I was given.	9	4.0
Information I learned by attending a medical conference	8	3.6
Unknown	7	3.1
Television	2	0.9
Abnormal laboratory test	1	0.4
Other	1	0.4
**Total**	**223**	**100**

[a]104 (51.0%) of 204 patients reported a mean of 2.1±1.4 different motivations to seek a further diagnosis. 59 (56.7%) reported more than 1. 100 patients did not receive other non-mitochondrial disease diagnoses. 11 patients who did not respond are excluded.

S8 Table. Changes (N, %) in disease management or treatment after mitochondrial disease diagnosis (441 changes from 149 patients), Panel A; and after genetic confirmation of mitochondrial disease diagnosis (184 changes from 65 patients), Panel B.

	N	%
**A Changes in disease management or treatment after mitochondrial disease diagnosis[a]**		
Nutritional supplements changed	115	26.1
Additional diagnostic tests ordered (e.g. blood test, EKG, brain MRI, echocardiogram, etc.)	114	25.9
Medications changed	99	22.4
Exercise therapy instituted	46	10.4
Dietary therapy started	45	10.2
Lifestyle changes	8	1.8
Other specific symptom related therapy	6	1.4
Referral to new specialists	4	0.9
Surgery	3	0.7
Clinical trial	1	0.2
Other	0	0.0
**Total**	**441**	**100**
[a]149 (72.3%) of 206 patients reported a mean of 3.0±1.3 changes after mitochondrial diagnosis. 128 (85.9%) reported more than one, 57 reported no change. 9 patients who did not respond are excluded.		
**B Changes in disease management or treatment after genetic confirmation of mitochondrial disease diagnosis[b]**		
Additional diagnostic tests ordered (e.g. blood test, EKG, brain MRI, echocardiogram, etc.)	45	24.5
Nutritional supplements changed	45	24.5
Medications changed	44	23.9
Exercise therapy instituted	20	10.9
Dietary therapy started	19	10.3
Other specific symptom related therapy	3	1.6
Clinical trial	2	1.1
Lifestyle changes	2	1.1
Referral to new specialists	2	1.1
Unknown	2	1.1
**Total**	**184**	**100**
[b]65 (32.2%) of 202 patients received genetic confirmation and had a change in disease management or treatment. Of these, 64 (98.5%) reported a mean of 2.9±1.4 changes after the confirmation, 51 (78.5%) reported more than one change. 72 received genetic confirmation and reported no change. 65 patients reported no genetic confirmation. 13 patients who did not respond are excluded.		

S9 Table. Changes (N, %) in health care team after a mitochondrial disease diagnosis. N=151 responses from 98 patients.[a]

Change	N	%
Referred to another specialist	81	53.6
Referred to a patient advocacy group	49	32.5
Referred to a support group	21	13.9
**Total**	**151**	**100**

[a]98 (47.6%) of 206 patients reported a mean of 1.6$\pm$0.7 changes, 42 (42.9%) reported more than 1, and 108 reported no change. 9 patients who did not respond are excluded.

S10 Table. Groups (N, %) joined after mitochondrial disease diagnosis. N=281 responses from 151 patients.[a]

Group	N	%
Facebook group	102	36.3
Patient support	76	27.0
Patient advocacy	73	26.0
Online message board	28	10.0
Patient registry	1	0.4
Unknown	1	0.4
**Total**	**281**	**100**

[a]151 (74%) of 204 of patients reported joining a mean of 1.9$\pm$1.0 patient support groups. 80 (53.0%) reported joining more than 1. 53 did not join any group. 11 patients who did not respond are excluded.

S11 Table. Benefits (N, %) of joining groups. N=188 responses from 122 patients.[a]

Benefit	N	%
Develop connections with other patients and families, developing a sense of community	56	29.8
Improved education	43	22.9
Get advice and practical tips from other patients and families	35	18.6
Support	21	11.2
Validation of one's symptoms	15	8.0
Learning about ongoing research	7	3.7
Raising awareness about the disease	6	3.2
Empowered to lead a support group	3	1.6
Unknown	2	1.1
**Total**	**188**	**100**

[a]122 (81.3%) of 150 patients reported a mean of 1.7±0.87 benefits from joining patient support groups. 57 (46.7%) reported more than1, 28 did not report any. 65 patients who did not respond are excluded.

S12 Table. Anticipated impact if mitochondrial diagnosis is wrong. N=200 patients.[a]

Response	N	%
Very negative	38	19.0
Negative	52	26.0
No effect at all	57	28.5
Positive	29	14.5
Very Positive	24	12.0
**Total**	**200**	**100**

[a]15 patients did not respond.
