# Supplementary figures and images for: The evolution of the mitochondrial disease diagnostic odyssey
Source: Orphanet J Rare Dis. 2023 Jun 22;18:157. doi: 10.1186/s13023-023-02754-x (PMC10288668; doi:10.1186/s13023-023-02754-x)

S1 Fig.

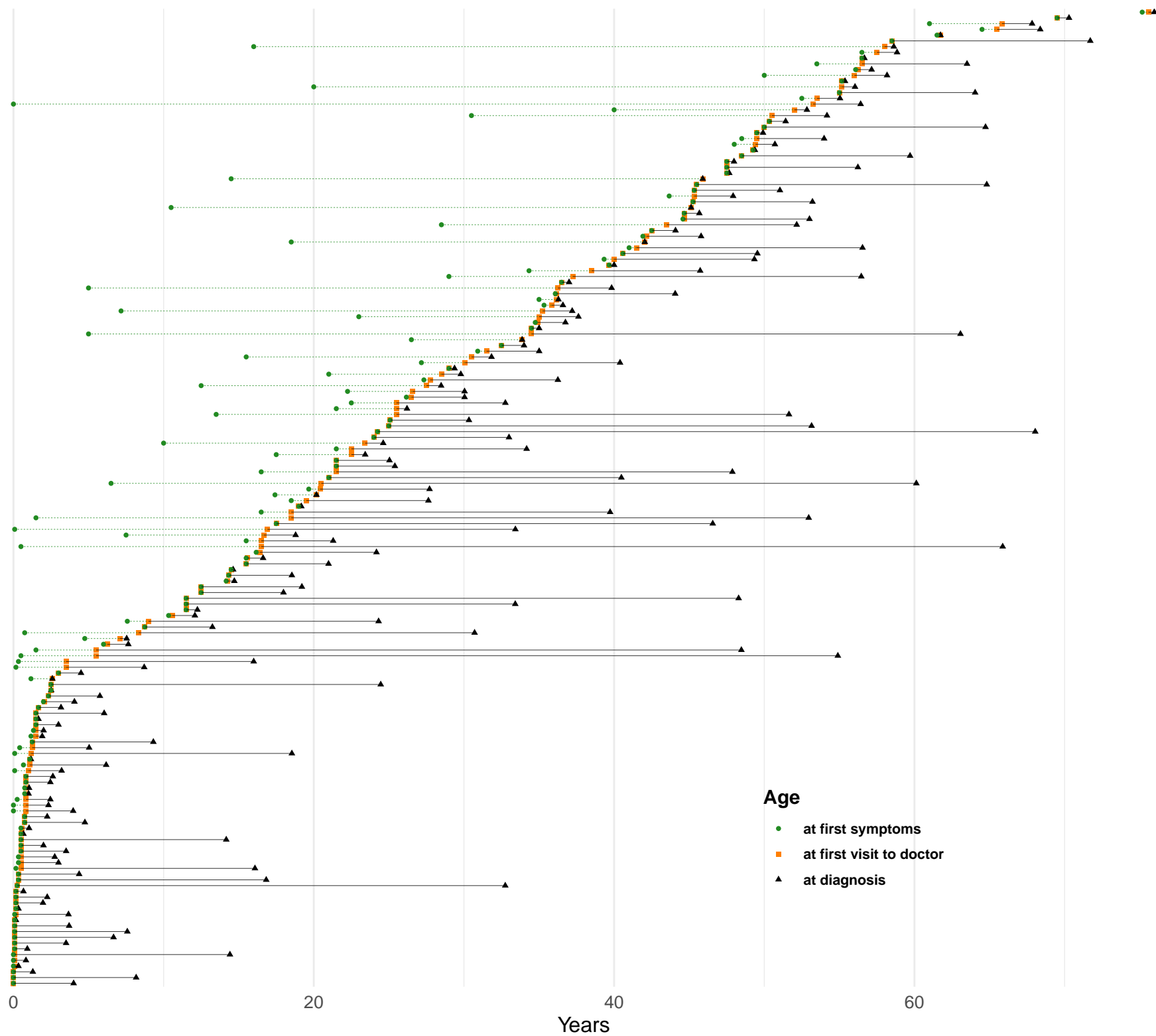

**S2 Fig.**

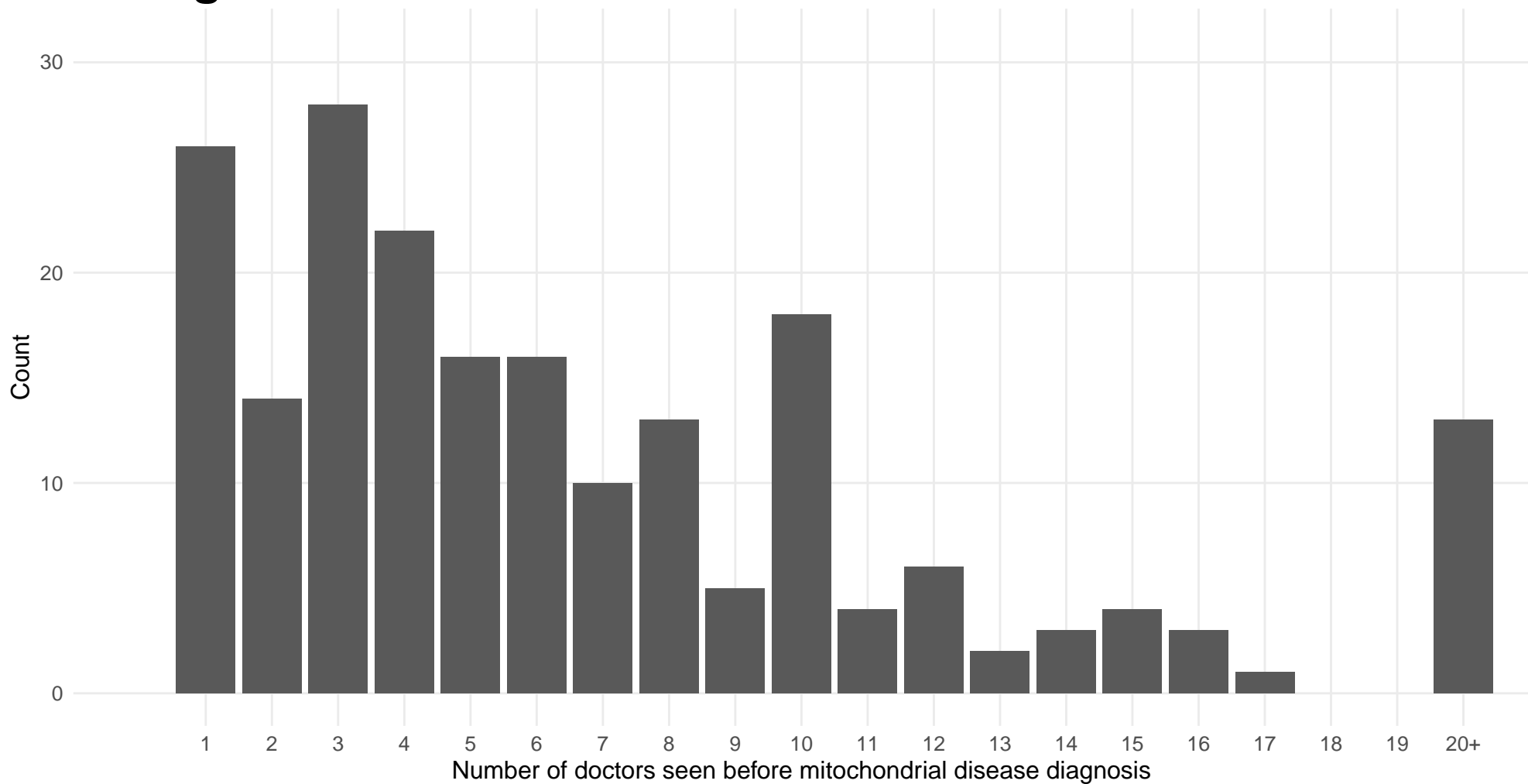

Supplement: Supplementary file 2 — Additional file 2: Figure S1. Individual patient trajectories, from age in years at first symptoms of mitochondrial disease, through age at first visit to doctor, to age at diagnosis, sorted by age at first visit to doctor. 45 cases with missing or contradictory information are omitted.. Figure S2. Distribution of Number of Doctors Seenby patients with mitochondrial disease from symptom onset to diagnosis.. [file 13023_2023_2754_MOESM2_ESM.pdf]
