# Supplementary material for: The evolution of the mitochondrial disease diagnostic odyssey
Source: Orphanet J Rare Dis. 2023 Jun 22;18:157. doi: 10.1186/s13023-023-02754-x (PMC10288668; doi:10.1186/s13023-023-02754-x)
Supplement: Supplementary file 3 — Additional file 3. The Diagnostic Odyssey 2 Survey Questionnaire. [file 13023_2023_2754_MOESM3_ESM.pdf]

Survey Instrument for Odyssey 2 (RDCRN/NAMDC + UMDF)

BEFORE PROCEEDING: If you are a member of both the RDCRN Contact Registry and the UMDF MDCR Registry you may receive two copies of this survey, both sent out on the same day. They are identical. Please COMPLETE ONLY ONE. THIS IS VERY IMPORTANT.

Q0: Have you already completed this Odyssey2 survey?

- *YES. I have previously completed the Odyssey2 survey.*
- *NO. This is my first time completing the Odyssey2 survey.*

Mitochondrial diseases are difficult to diagnose. This survey will update our knowledge of patients' experiences as they seek a mitochondrial disease diagnosis, so that we can improve the process.

You can answer the questions for yourself, your child, or someone for whom you are the caregiver, so long as a confirmed mitochondrial disease diagnosis has been received from a doctor. If you are responding for someone else, please read the words 'you' and 'your' as applying to the patient, with the exception of Question 1.

If you have not been informed by a doctor that you have a confirmed mitochondrial disorder, thank you for your interest, but do not proceed with the survey.

Q1: Are you completing this survey for yourself, or for someone else?

- Yourself
- Your child
- Someone else, for whom you are a caregiver
- Other:
  Please specify: [text box]

All questions below are about the person affected by mitochondrial disease for whom this survey is intended.

Q2: What is your age? *If possible, please give both years and months. Example: 1 year, 4 months. Months are especially important for very young children (less than 2 years old).*

[Drop down lists 0-99 for years and 0-11 for months]

Q3: Have you been informed by a doctor that you have mitochondrial disease?

- Yes
- No

Q4: What is your confirmed mitochondrial diagnosis?

- o Alpers-Huttenlocher syndrome (Alpers syndrome)
- o Aminoglycoside-induced deafness
- o Barth syndrome (lethal infantile cardiomyopathy)
- o CPEO (Chronic progressive external ophthalmoplegia) or CPEO “plus”
- o Coenzyme Q10 deficiency
- o Maternally Inherited Diabetes and deafness (MIDD)
- o Encephalomyopathy
- o Encephalopathy
- o Hepatocerebral syndrome
- o Kearns-Sayre syndrome
- o Leber hereditary optic neuropathy (LHON)
- o Leigh syndrome
- o Leukodystrophy
- o Mitochondrial encephalomyopathy, lactic acidosis, and stroke-like episodes (MELAS)
- o Mitochondrial neurogastrointestinal encephalopathy (MNGIE)
- o Multi-systemic syndrome
- o Myoclonic epilepsy with ragged-red fibers (MERRF)
- o Mitochondrial myopathy
- o Mitochondrial DNA (mtDNA) depletion syndrome
- o Mitochondrial DNA (mtDNA) deletion(s)
- o Neuropathy, ataxia, and retinitis pigmentosa (NARP)
- o Pearson syndrome
- o Reversible infantile myopathy with cytochrome c oxidase deficiency
- o Polymerase gamma (POLG) related disorders/ataxia neuropathy spectrum
- o Pyruvate dehydrogenase deficiency (PDH)
- o Pyruvate carboxylase deficiency (PC)
- o Other

Please specify: [text box]

Q5: Have you been <u>informed by a doctor</u> that you have a “biochemical deficiency” (or “biochemical diagnosis”)?

- o Yes [answer Q5A]
- o No [move on to Q6]

Q5A: What biochemical deficiency (or biochemical diagnosis) were you told that you have? You may select more than one deficiency or diagnosis.

- ☐ Carnitine deficiency
- ☐ Coenzyme Q10 deficiency
- ☐ Complex I deficiency

☐ Complex II deficiency
☐ Complex III deficiency
☐ Complex IV deficiency
☐ Complex V deficiency
☐ Multiple complex deficiency
☐ Oxidative phosphorylation (OXPHOS) deficiency
☐ Fatty acid oxidation defect
☐ Loose coupling of oxidation and phosphorylation
☐ Pyruvate dehydrogenase complex (PDC) deficiency
☐ Thymidine phosphorylase deficiency
☐ Other
Please specify [text box]

Q6: To your best recollection, what was the first symptom (or symptoms) that you noticed and would attribute to the onset of your mitochondrial disease? You may select more than one symptom, but **only** if they occurred around the same time.

☐ Change in mental health
☐ Developmental delay
☐ Diabetes
☐ Difficulty walking
☐ Droopy eyelids
☐ Failure to grow or gain weight
☐ Fatigue
☐ Floppiness/hypotonia
☐ Gastrointestinal discomfort or dysfunction
☐ Hearing loss
☐ Heart disease
☐ Impaired coordination
☐ Kidney disease
☐ Liver disease
☐ Loss of vision
☐ Numbness, weakness or both in your hands and/or feet
☐ Seizures
☐ Weakness
☐ Other:
Please specify [text box]

Q7: How old were you when you developed this first symptom or symptoms?

[Drop down lists 0-99 for years and 0-11 for months]

Q8: What symptom or symptoms motivated you to see a doctor for the first time? This may or may not be the same as the first symptom(s) you noticed (Q6). You may select more than one symptom**.**

- ☐ Change in mental health
- ☐ Developmental delay
- ☐ Diabetes
- ☐ Difficulty walking
- ☐ Droopy eyelids
- ☐ Failure to grow or gain weight
- ☐ Fatigue
- ☐ Floppiness/hypotonia
- ☐ Gastrointestinal discomfort or dysfunction
- ☐ Hearing loss
- ☐ Heart disease
- ☐ Impaired coordination
- ☐ Kidney disease
- ☐ Liver disease
- ☐ Loss of vision
- ☐ Numbness, weakness, or both in your hands and/or feet
- ☐ Seizures
- ☐ Weakness
- ☐ Other

  Please specify [text box]

Q9: How old were you when you first visited this doctor (with whom you first discussed symptoms)?

[Drop down lists 0-99 for years and 0-11 for months]

Q10: Was this doctor (with whom you first discussed symptoms)

- o your primary care physician? [move on to Q11]
- o a specialist? [move on to Q10A]

Q10A: What type of specialist was the doctor (with whom you first discussed symptoms)?

- o Cardiologist
- o Clinical Geneticist
- o Ear, Nose, and Throat Specialist (Otolaryngologist)
- o Endocrinologist
- o Infectious Disease
- o Gastroenterologist
- o Metabolic Disease Specialist
- o Neurologist
- o Neuromuscular Specialist
- o Ophthalmologist
- o Pulmonologist
- o Rheumatologist

- Other
  Please specify [text box]

Q11: Was this doctor (with whom you first discussed your symptoms) the one <u>who eventually diagnosed your mitochondrial disease</u>?

- Yes [move on to Q13]
- No [answer Q11A]

Q11A: Approximately how many doctors did you discuss your symptoms with? <u>Include</u> the one you first discussed your symptoms with, <u>and</u> the one who eventually diagnosed your mitochondrial disease; <u>and</u> any others.

[drop down list 1-20]

Q12: What was the specialty of the doctor who eventually diagnosed your mitochondrial disease?

- Cardiologist
- Clinical Geneticist
- Ear, Nose, and Throat Specialist (ENT, Otolaryngologist)
- Endocrinologist
- Infectious Disease
- Gastroenterologist
- Metabolic Disease Specialist
- Neurologist
- Neuromuscular Specialist
- Ophthalmologist
- Pulmonologist
- Rheumatologist
- Other
  Please specify [text box]

Q13: When did you receive this mitochondrial disease diagnosis? Please provide month and year.

[Drop down lists for year (1950-2018) and month (01-12)]

Q14: Was this doctor (who eventually diagnosed your mitochondrial disease) considered a recognized mitochondrial disease expert?

- Yes
- No

Q15: From the time you saw the first doctor (the one with whom you first discussed your symptoms), how long did it take to get a confirmed mitochondrial disease diagnosis?

[drop down lists for years (0-99) and months (0-11)]

Q16: Between the time when you first discussed your symptoms with a doctor and the time that you received a mitochondrial disease diagnosis, did you receive any other, non-mitochondrial disease diagnoses that you were told were the cause of your symptoms?

- o Yes [answer Q16A and Q16B]
- o No [move on to Q17]

Q16A: What non-mitochondrial disease diagnosis or diagnoses did you receive? You can check more than one diagnosis.

☐ Psychiatric disorder
☐ Fibromyalgia
☐ Chronic fatigue syndrome
☐ Multiple sclerosis
☐ Gastrointestinal disease
☐ Seizure
☐ Myasthenia gravis
☐ Rheumatological disorder
☐ Chronic pain
☐ Other
Please specify [text box]

Q16B: After receiving this non-mitochondrial disease diagnosis, what motivated you to seek out a different diagnosis? You can select more than one reason.

☐ My symptoms were not improving with treatment.
☐ Consultation(s) with other doctors.
☐ Consultation with people not in the medical profession
☐ Information I learned by attending a medical conference.
☐ Web search or browsing.
☐ Television.
☐ Reading magazine(s), journal, or other print media.
☐ Information from a patient group.
☐ Other
Please specify [text box]

Q17: Between the time when you first discussed your symptoms with a doctor and the time that you received a mitochondrial disease diagnosis, how many health care professionals were on your care team?

[drop down list 1-20]

Q17A: Between the time when you first discussed your symptoms with a doctor and the time that you received a mitochondrial disease diagnosis, what were the specialties of the members of your care team?

- o Cardiologist
- o Clinical Geneticist
- o Ear, Nose, and Throat Specialist (ENT, Otolaryngologist)
- o Endocrinologist
- o Infectious Disease
- o Gastroenterologist
- o Metabolic Disease Specialist
- o Neurologist
- o Neuromuscular Specialist
- o Ophthalmologist
- o Pulmonologist
- o Rheumatologist
- o Other

    Please specify [text box]

Q18: As a result of receiving a mitochondrial disease diagnosis, has your disease management or treatment changed?

- o Yes [answer Q18A]
- o No [move on to Q19]

Q18A: If yes, how did your disease management or treatment change? You may select more than one change.

☐ Medications changed
☐ Nutritional supplements changed
☐ Exercise therapy instituted
☐ Dietary therapy started
☐ Additional diagnostic tests ordered (e.g. blood test, EKG, brain MRI, echocardiogram, etc.)
☐ Other

    Please specify [text box]

Q19: As a result of receiving a genetic confirmation for your mitochondrial disease diagnosis, has your disease management or treatment changed?

- Yes [answer Q19A]
- No [move on to Q20]
- N/A – Did not receive genetic confirmation [move on to Q20]

Q19A: If yes, how did your disease management or treatment change? You may select more than one change.

☐ Medications changed
☐ Nutritional supplements changed
☐ Exercise therapy instituted
☐ Dietary therapy started
☐ Additional diagnostic tests ordered (e.g. blood test, EKG, brain MRI, echocardiogram, etc.)
☐ Other
Please specify [text box]

Q20: Did your health care team change as a result of receiving a mitochondrial disease diagnosis?

- Yes [answer Q20A]
- No [move on to Q21]

Q20A: If yes, how did your health care team change? You may select more than one change.

☐ Referred to another specialist(s)
Please select specialists. You may select more than one specialist.
☐ Neurologist
☐ Clinical Geneticist
☐ Metabolic Disease Specialist
☐ Mitochondrial Disease Specialist
☐ Referred to a support group
☐ Referred to a patient advocacy group such as United Mitochondrial Disease Foundation (UMDF), Muscular Dystrophy Association (MDA), or MitoAction

Q21: Do you feel that having a mitochondrial disease diagnosis changed health professionals' perception of you or your complaints?

- Yes
  Please explain [text box]
- No

Q22: Before your diagnosis was confirmed, do you think that NOT having a mitochondrial disease diagnosis influenced the way doctors treated you?

- Yes

- No

Q23: As a result of receiving a mitochondrial disease diagnosis, did you join a patient support group, patient advocacy group, Facebook group, online message board, or any other sort of community for individuals with mitochondrial disease?

- Yes [Answer Q23A and Q23B]
- No [Move on to Q24]

Q23A: Which type of group(s) did you join? You may select more than one group.

☐ Patient support group
☐ Patient advocacy group
☐ Facebook group
☐ Online message board
☐ Other: Please specify

Q23B: Do you feel participation and/or membership in such a community has been beneficial?

- Yes [Answer Q23C]
- No [Move on to Q24]

Q23C: What have been the benefits?

Please specify [text box]

Q24: If you were to learn today that the mitochondrial disease diagnosis you received is incorrect, and that you do not in fact have a mitochondrial disease, how would this affect you?

- It would affect me very negatively.
- It would affect me negatively.
- It would not affect me at all.
- It would affect me positively.
- It would affect me very positively.
